# The nuclease activity of DNA2 promotes exonuclease 1–independent mismatch repair

**DOI:** 10.1016/j.jbc.2022.101831

**Published:** 2022-03-15

**Authors:** Lyudmila Y. Kadyrova, Basanta K. Dahal, Vaibhavi Gujar, James M. Daley, Patrick Sung, Farid A. Kadyrov

**Affiliations:** 1 Department of Biochemistry and Molecular Biology, Southern Illinois University School of Medicine, Carbondale, Illinois, USA; 2 Department of Biochemistry and Structural Biology, University of Texas Health Science Center, San Antonio, Texas, USA

**Keywords:** EXO1, exonuclease 1, MMR, DNA mismatch repair, Pol δ, DNA polymerase δ, Pol ε, DNA polymerase ε, PCNA, proliferating cell nuclear antigen, RFC, replication factor C, RPA, replication protein A

## Abstract

The DNA mismatch repair (MMR) system is a major DNA repair system that corrects DNA replication errors. In eukaryotes, the MMR system functions *via* mechanisms both dependent on and independent of exonuclease 1 (EXO1), an enzyme that has multiple roles in DNA metabolism. Although the mechanism of EXO1-dependent MMR is well understood, less is known about EXO1-independent MMR. Here, we provide genetic and biochemical evidence that the DNA2 nuclease/helicase has a role in EXO1-independent MMR. Biochemical reactions reconstituted with purified human proteins demonstrated that the nuclease activity of DNA2 promotes an EXO1-independent MMR reaction *via* a mismatch excision-independent mechanism that involves DNA polymerase δ. We show that DNA polymerase ε is not able to replace DNA polymerase δ in the DNA2-promoted MMR reaction. Unlike its nuclease activity, the helicase activity of DNA2 is dispensable for the ability of the protein to enhance the MMR reaction. Further examination established that DNA2 acts in the EXO1-independent MMR reaction by increasing the strand-displacement activity of DNA polymerase δ. These data reveal a mechanism for EXO1-independent mismatch repair.

The mismatch repair (MMR) system has been conserved from bacteria to humans (1, 2). It promotes genome stability by suppressing spontaneous and DNA damage-induced mutations (1, 3, 4, 5, 6, 7, 8, 9, 10, 11). The key function of the MMR system is the correction of DNA replication errors that escape the proofreading activities of replicative DNA polymerases (1, 4, 5, 6, 7, 8, 9, 10, 12). In addition, the MMR system removes mismatches formed during strand exchange in homologous recombination, suppresses homeologous recombination, initiates apoptosis in response to irreparable DNA damage caused by several anticancer drugs, and contributes to instability of triplet repeats and alternative DNA structures (1, 4, 5, 7, 8, 9, 10, 11, 13, 14, 15, 16, 17, 18). The principal components of the eukaryotic MMR system are MutSα (MSH2-MSH6 heterodimer), MutLα (MLH1-PMS2 heterodimer in humans and Mlh1-Pms1 heterodimer in yeast), MutSβ (MSH2-MSH3 heterodimer), proliferating cell nuclear antigen (PCNA), replication factor C (RFC), exonuclease 1 (EXO1), RPA, and DNA polymerase δ (Pol δ). Loss-of-function mutations in the *MSH2*, *MLH1*, *MSH6*, and *PMS2* genes of the human MMR system cause Lynch and Turcot syndromes, and hypermethylation of the *MLH1* promoter is responsible for ∼15% of sporadic cancers in several organs (19, 20). MMR deficiency leads to cancer initiation and progression *via* a multistage process that involves the inactivation of tumor suppressor genes and action of oncogenes (21).

MMR occurs behind the replication fork (22, 23) and is a major determinant of the replication fidelity (24). The correction of DNA replication errors by the MMR system increases the replication fidelity by ∼100 fold (25). Strand breaks in leading and lagging strands as well as ribonucleotides in leading strands serve as signals that direct the eukaryotic MMR system to remove DNA replication errors (26, 27, 28, 29, 30). MMR is more efficient on the lagging than the leading strand (31). The substrates for MMR are all six base–base mismatches and 1 to 13-nt insertion/deletion loops (25, 32, 33, 34). Eukaryotic MMR commences with recognition of the mismatch by MutSα or MutSβ (32, 34, 35, 36). MutSα is the primary mismatch-recognition factor that recognizes both base–base mismatches and small insertion/deletion loops whereas MutSβ recognizes small insertion/deletion loops (32, 34, 35, 36, 37). After recognizing the mismatch, MutSα or MutSβ cooperates with RFC-loaded PCNA to activate MutLα endonuclease (38, 39, 40, 41, 42, 43). The activated MutLα endonuclease incises the discontinuous daughter strand 5′ and 3′ to the mismatch. A 5' strand break formed by MutLα endonuclease is utilized by EXO1 to enter the DNA and excise a discontinuous strand portion encompassing the mismatch in a 5'→3′ excision reaction stimulated by MutSα/MutSβ (38, 44, 45). The generated gap is filled in by the Pol δ holoenzyme, and the nick is ligated by a DNA ligase (44, 46, 47). DNA polymerase ε (Pol ε) can substitute for Pol δ in the EXO1-dependent MMR reaction, but its activity in this reaction is much lower than that of Pol δ (48). Although MutLα endonuclease is essential for MMR *in vivo*, 5′ nick-dependent MMR reactions reconstituted in the presence of EXO1 are MutLα-independent (44, 47, 49).

EXO1 deficiency in humans does not seem to cause significant cancer predisposition (19). Nevertheless, it is known that Exo1^-/-^ mice are susceptible to the development of lymphomas (50). Genetic studies in yeast and mice demonstrated that EXO1 inactivation causes only a modest defect in MMR (50, 51, 52, 53). In agreement with these genetic studies, a defined human EXO1-independent MMR reaction that depends on the strand-displacement DNA synthesis activity of Pol δ holoenzyme to remove the mismatch was reconstituted (54). Furthermore, an EXO1-independent MMR reaction that occurred in a mammalian cell extract system without the formation of a gapped excision intermediate was observed (54). Together, these findings implicated the strand-displacement activity of Pol δ holoenzyme in EXO1-independent MMR.

In this study, we investigated DNA2 in the context of MMR. DNA2 is an essential multifunctional protein that has nuclease, ATPase, and 5'→3′ helicase activities (55, 56, 57). Previous research ascertained that DNA2 removes long flaps during Okazaki fragment maturation (58, 59, 60), participates in the resection step of double-strand break repair (61, 62, 63), initiates the replication checkpoint (64), and suppresses the expansions of GAA repeats (65). We have found *in vivo* and *in vitro* evidence that DNA2 promotes EXO1-independent MMR. Our data have indicated that the nuclease activity of DNA2 enhances the strand-displacement activity of Pol δ holoenzyme in an EXO1-independent MMR reaction.

## Results

### A *dna2* allele causes a defect in Exo1-independent MMR

**Figure 1:**
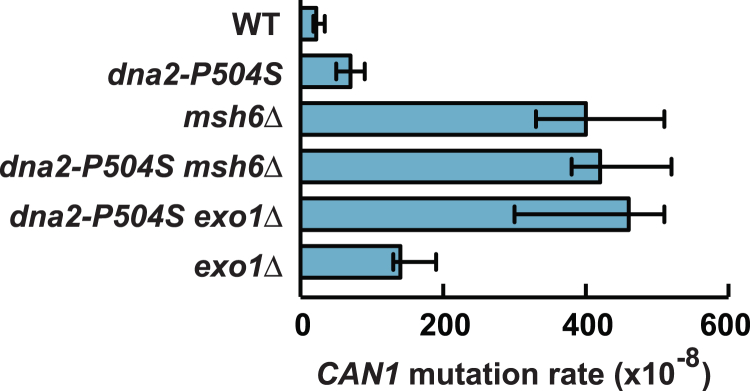
**A synergistic interaction between *dna2-P504S* and *exo1Δ* for *can1* mutations.** Spontaneous *CAN1* mutation rates were measured as described under Experimental procedures. The data are presented as medians with 95% confidence intervals.

We started this work to investigate whether DNA2 has a role in MMR. Our initial genetic analysis in *Saccharomyces cerevisiae* demonstrated that introduction of a temperature-sensitive *dna2* allele (55), *dna2-P504S*, into a WT strain caused a 3-fold increase in the *CAN1* mutation rate (Fig. 1). We then used the *CAN1* mutation assay to examine how *dna2-P504S* interacted with *exo1Δ* and *msh6Δ*, two alleles that cause MMR defects. The data showed that there was a synergistic relationship between *dna2-P504S* and *exo1Δ*, but not between *dna2-P504S* and *msh6Δ* (Fig. 1). Next, we determined *can1* mutation spectra in the WT, *dna2-P504S*, *exo1Δ*, *dna2-P504S exo1Δ, msh2Δ*, and *msh2Δ dna2-P504S exo1Δ* strains (Table 1). The *can1* spectrum in the *dna2-P504S* strain was dominated by base–base substitutions, but some 1-nt deletions and mutational events that caused *CAN1* loss were also observed. Comparison of the mutation spectra in the *dna2-P504S*, *exo1Δ*, and *dna2-P504S exo1Δ* showed that there was a synergistic interaction between *dna2-P504S* and *exo1Δ* for base substitutions and 1-nt deletions. Further analysis revealed that *msh2Δ* was epistatic to *dna2-P504S exo1Δ* for base substitutions. The observations that (i) *msh2Δ* was epistatic to *dna2-P504S exo1Δ* for base substitutions and that (ii) there was a synergistic relationship between *dna2-P504S* and *exo1Δ* for base substitutions supported the hypothesis that *DNA2* is involved in an Msh2-dependent pathway that repairs base–base mismatches in an Exo1-independent manner. We also observed that there was a weak synergistic relationship between *msh2Δ* and *dna2-P504S exo1Δ* for 1-nt deletions. This is likely a result of participation of *DNA2* in another genetic stabilization pathway.

**Table 1:** *msh2Δ* is epistatic to *dna2-P504S exo1Δ* for base substitutions in *CAN1*

Genotype	Mutation rate (× 10^−8^)					
	Base–base substitutions	1-nt deletions	1-nt insertions	*CAN1* loss	Other mutations	Total
WT (n=50)	20	1.5	1.5	<0.5	1.5	24 (21–34)
*dna2*-*P504S* (n=50)	42	8	<2	8	11	70 (50–90)
*exo1Δ* (n=50)	110	14	<2.8	<2.8	14	140 (130–190)
*dna2-P504S exo1Δ* (n=80)	280	69	29	23	63	460 (300–510)
*msh2Δ* (n=82)	435	390	90	<11	<11	910 (900–1200)
*msh2Δ dna2-P504S exo1Δ* (n=79)	425	655	61	61	<15	1200 (1100–1700)
*pms1-Q723A* (n=51)	85	36	11	3	3	140 (110–180)
*dna2-P504S pms1-Q723A* (n=50)	80	110	35	5	20	250 (230–320)

n, a number of *can1* mutants sequenced. The mutations were identified by DNA sequencing. 95% confidence intervals are in parentheses.

A physical interaction between MutLα and PCNA is essential for MMR (66). A mutation, *pms1-Q723A*, that results in an amino acid change in the PCNA-binding motif of MutLα causes a strong defect in EXO1-independent MMR (66). We investigated how *pms1-Q723A* and *dna2-P504S* interacted with each other in the *CAN1* mutation assay. Measurements of the mutation rates and analysis of the mutation spectra revealed that *pms1-Q723A* was epistatic to *dna2-P504S* for base substitutions in *CAN1* (Table 1). This finding provided additional genetic evidence for a role of DNA2 in EXO1-independent MMR.

### DNA2 promotes an EXO1-independent MMR reaction in a reconstituted system *via* an excision-independent mechanism

**Figure 2:**
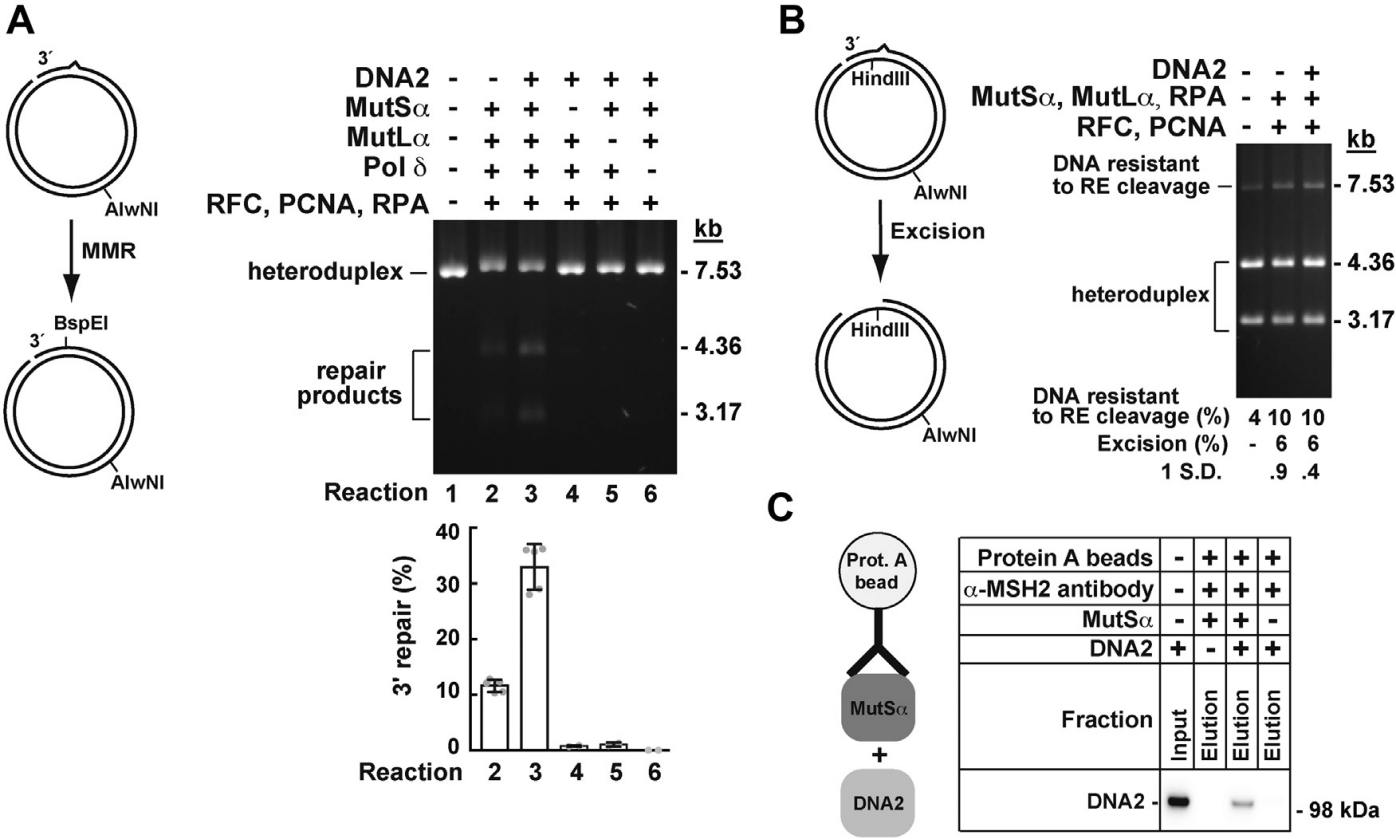
**DNA2 promotes EXO1-independent MMR on a 3′ heteroduplex and physically interacts with MutSα.** Reconstituted human MMR (*A*) and mismatch excision (*B*) reactions were carried out for 20 min at 37 °C as detailed under Experimental procedures. The DNA substrate for the reactions contained a 21-nt gap 304-bp 3′ to an A-C mispair. When indicated, the reaction mixture contained MutSα (25 nM), MutLα (10 nM), DNA2 (15 nM), PCNA (30 nM), RFC (10 nM), RPA (10 nM), and Pol δ (10 nM). To score MMR, the reaction products were cleaved with BspEI and AlwNI. *A*, an outline of the MMR assay is shown on the *left*. In this 3′ MMR assay, a strand break-directed repair of the A-C mispair leads to restoration of a BspEI site. The gel image shows MMR products that were formed in the presence of indicated proteins. The data in the graph were obtained by quantification of gel images like the one shown and are presented as averages ±1 S.D. (n > 3). *B*, a sketch of the mismatch excision assay is depicted on the *left*. In this assay, a strand break-directed excision of the A-C mispair leads to the formation of a gap that renders the DNA resistant to cleavage with HindIII. A gel image on the *right* shows mismatch excision products that were generated in the presence indicated proteins. The excision values were calculated by subtracting percentages of the uncleaved/gapped species that were observed in reactions 2 and 3 from percentage of the uncleaved/gapped species that was observed in the control reaction containing the substrate DNA only (reaction 1). The excision data are presented as averages ±1 S.D. (n = 4). *C*, a coimmunoprecipitation assay (Experimental procedures) was employed to detect a protein–protein interaction between purified MutSα and DNA2. When indicated, 30-μl reaction mixtures contained MutSα (3.4 pmol) and DNA2 (3.4 pmol). 0.4% of the input and 25% of the eluted fractions were analyzed by immunoblotting. A representative image is shown. MMR, DNA mismatch pair; Pol δ, DNA polymerase; PCNA, proliferating cell nuclear antigen; RFC, replication factor C; RPA, replication protein A.

We next utilized biochemical approaches to investigate DNA2 in the context of MMR reactions. A previous study described a defined EXO1-independent human MMR reaction that occurs on heteroduplex DNAs in the presence of MutSα, MutLα, PCNA, RFC, RPA, and Pol δ (54). In this EXO1-independent MMR reaction, MutLα incises a heteroduplex DNA 5′ to the mismatch in a MutSα-, PCNA-, and RFC-dependent manner, and Pol δ holoenzyme utilizes the 5′ strand break to perform a strand-displacement DNA synthesis that removes the mismatch. We examined whether DNA2 affected the EXO1-independent MMR reaction on a 3' heteroduplex DNA. Human DNA2 for these and following experiments was produced in and purified from insect Sf9 cells (57) (Fig. S1). The data revealed that the purified DNA2 increased the level of MMR in the EXO1-independent reaction by ∼3 fold (Fig. 2*A*, lanes 2–3 and graph). In the next series of experiments, we analyzed whether the presence of MutSα, MutLα, and Pol δ was necessary for the DNA2-promoted MMR reaction. As expected, these experiments showed that the omission of MutSα, MutLα, or Pol δ abolished the DNA2-promoted MMR reaction (Fig. 2*A*, lanes 4–6 and graph). Thus, we concluded that the DNA2-promoted MMR reaction on a 3′ heteroduplex occurred in a MutSα-, MutLα-, and Pol δ-dependent manner.

EXO1 excises the DNA mismatch in the mismatch excision reaction (45, 47, 67). To better understand the mechanism of DNA2-promoted MMR, we performed experiments to analyze whether the addition of DNA2 to a reaction mixture containing MutSα, MutLα, PCNA, RFC, RPA, and a 3′ heteroduplex led to a mismatch excision (Fig. 2*B*). In agreement with a previous study (38), a control experiment showed that a small level of mismatch excision took place in the reaction mixture containing MutSα, MutLα, PCNA, RFC, and RPA (Fig. 2*B*, lanes 1–2). This excision was a result of the activation of MutLα endonuclease by MutSα, PCNA, RFC, and the mismatch (38). However, we found that the supplementation of the five-protein system with DNA2 did not trigger an increase in the level of mismatch excision (Fig. 2*B*, lanes 2–3). This finding indicated that the mechanism of the DNA2-promoted MMR reaction is different from that of the EXO1-dependent MMR reaction.

Protein–protein interactions are involved in MMR reactions (4). We studied whether DNA2 physically interacted with the MMR factors MutSα and MutLα in a pull-down assay. The data showed that MutSα-containing agarose beads pulled down the purified DNA2 protein (Fig. 2*C*), but agarose beads containing MutLα did not (Fig. S2). Thus, these experiments revealed that DNA2 physically interacts with the mismatch recognition factor MutSα.

### DNA2 involves its nuclease activity to promote an EXO1-independent MMR reaction

**Figure 3:**
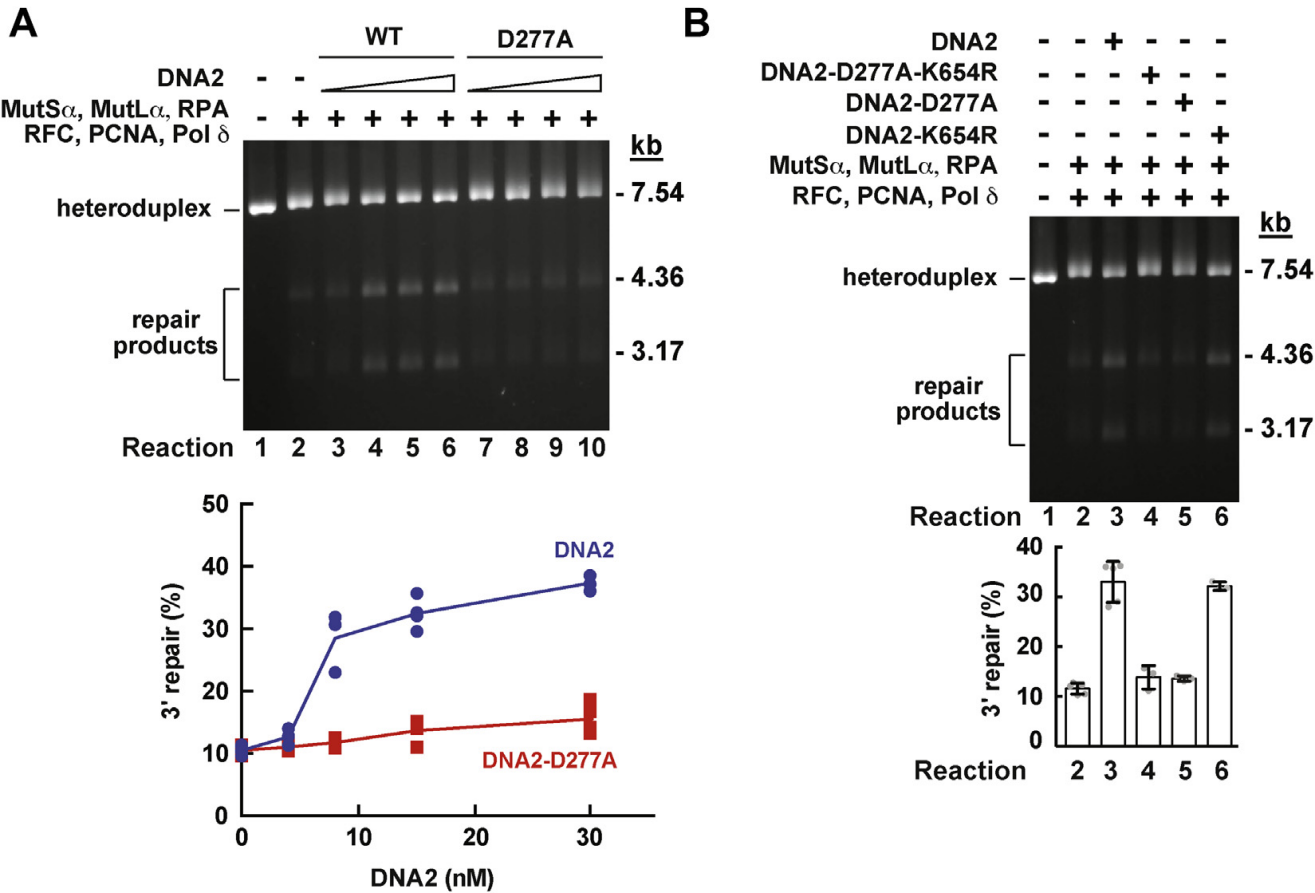
**The nuclease activity of DNA2 facilitates EXO1-independent MMR on a 3′ heteroduplex.** MMR reactions were carried out and analyzed as described in Fig. 2. *A*, the effects of different concentrations of DNA2 and the nuclease-deficient DNA2-D277A on EXO1-independent MMR on a 3′ heteroduplex. *B*, the effects of the helicase-deficient DNA2-K654R and the nuclease- and helicase-deficient DNA2-D277A-K654R on EXO1-independent MMR that occurred on a 3′ heteroduplex. The data in the graphs are averages ±1 S.D. (n > 3). MMR, DNA mismatch pair.

Human DNA2 has both helicase and nuclease activities (56, 57, 68). The DNA2-D277A variant lacks the nuclease activity and the DNA2-K654R mutant does not have the helicase activity (57). We investigated whether the DNA2-D277A and DNA2-K654R variants affected the EXO1-independent MMR reaction on a 3' heteroduplex DNA. We determined that the nuclease-deficient DNA2-D277A variant did not promote the 3′ gap-directed EXO1-independent MMR reaction (Fig. 3*A* and graph) but the helicase-deficient DNA2-K654R mutant protein did (Fig. 3*B*, lane 6 and graph). In agreement with the former result, we established the double mutant DNA2-D277A-K654R variant was not able to enhance the 3' gap-directed EXO1-independent MMR reaction. These findings indicated that DNA2 relies on its nuclease activity to promote the EXO1-independent MMR reaction on a 3′ heteroduplex DNA.

### DNA2 increases the strand-displacement DNA synthesis activity of the Pol δ holoenzyme

**Figure 4:**
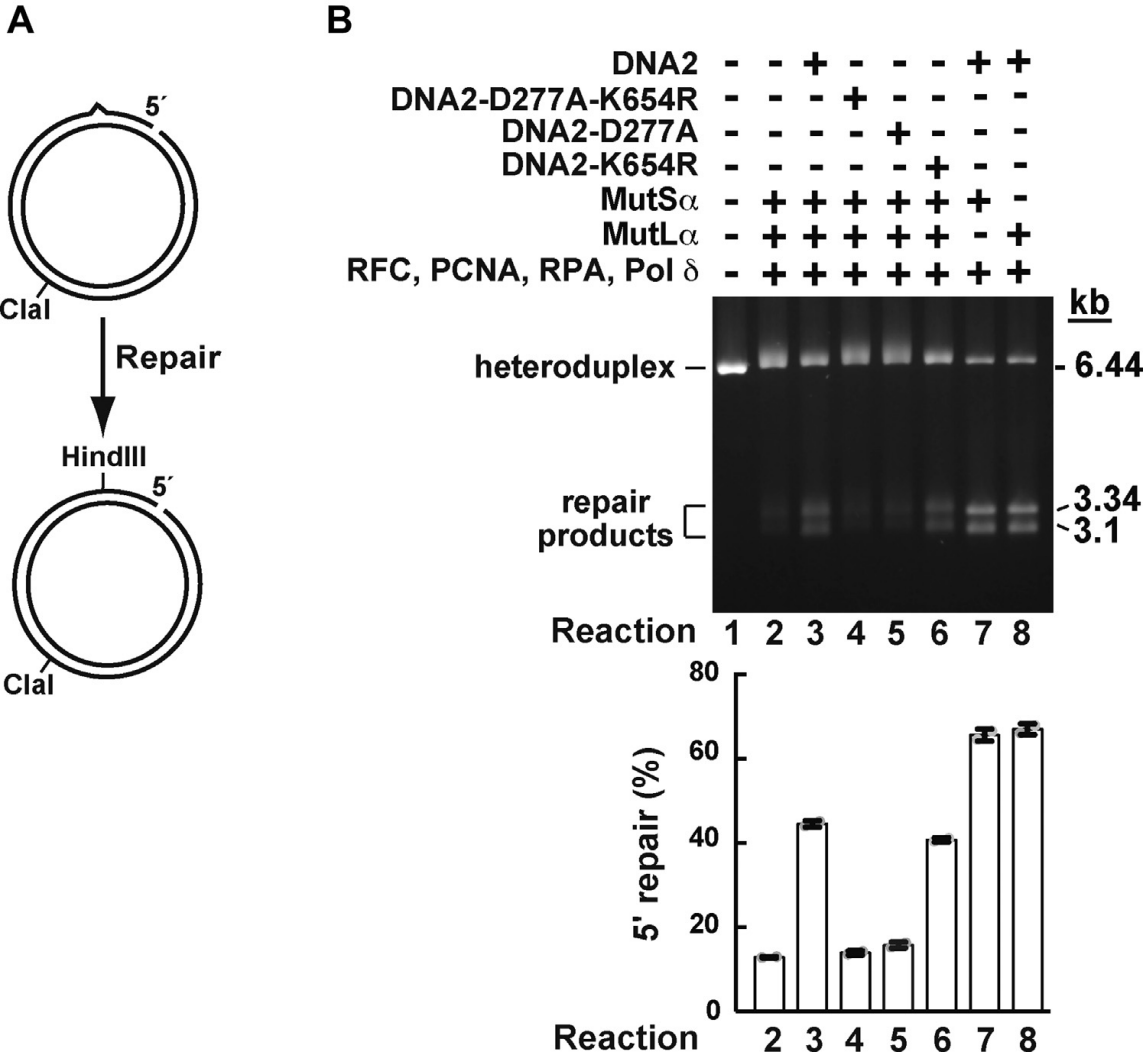
**The nuclease activity of DNA2 enhances EXO1-independent MMR on a 5′ heteroduplex.** MMR reactions were conducted as described in Fig. 2 except that the DNA substrate was a 5′ heteroduplex (a 5' G-T DNA), which carried a nick 128 bp 5′ to a G-T mispair. To score MMR, the reaction products were cleaved with HindIII and ClaI. *A*, a graphical representation of the 5′ MMR assay. *B*, MMR products that were generated in the presence of indicated proteins. The data in the graph were obtained by quantification of gel images including the one shown and are averages ±1 S.D. (n > 3). MMR, DNA mismatch pair.

**Figure 5:**
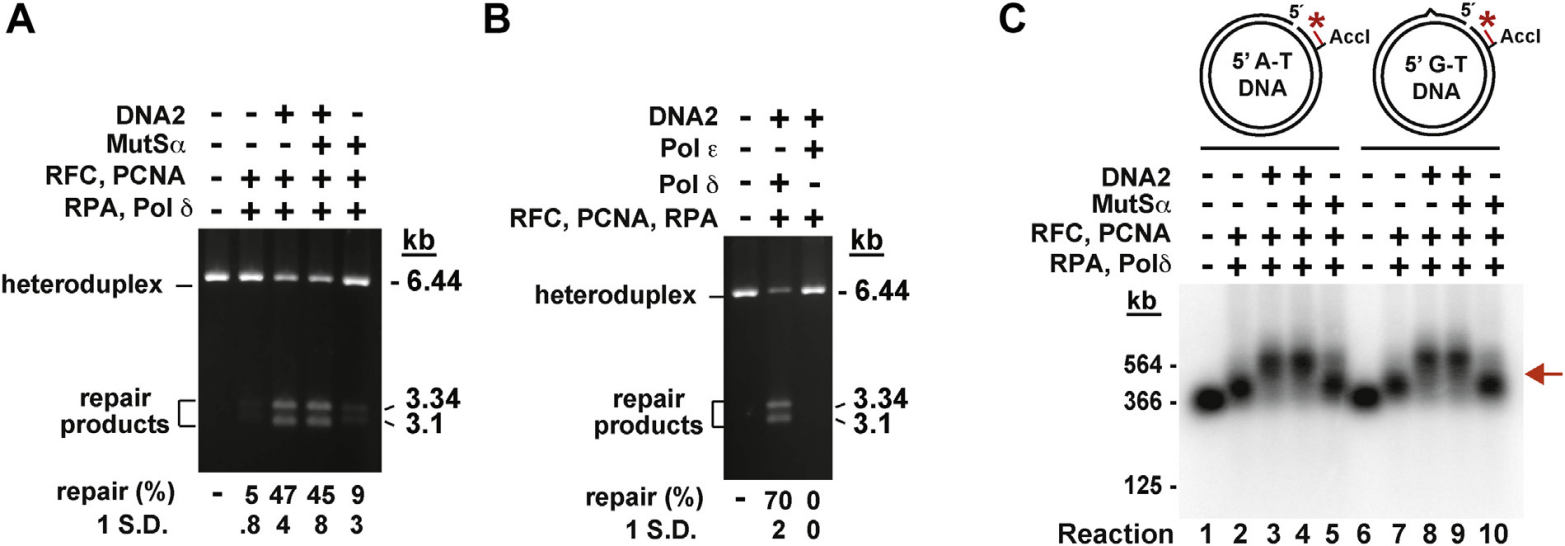
**DNA2 increases the strand-displacement activity of Pol δ holoenzyme.** MMR reactions on a 5' G-T DNA and control reactions on a 5′ A-T DNA were performed as described in Fig. 4 except that reactions in *A* and *C* were carried out for 10 min. *A*, the effect of DNA2 on the strand-displacement activity of Pol δ holoenzyme on a 5′ G-T DNA. *B*, the effect of replacement of Pol δ with Pol ε on the DNA2-promoted MMR on a 5′ G-T DNA. The data in *A* and *B* are averages ±1 S.D. (n > 3). *C*, a Southern hybridization analysis of strand-displacement products that were formed on 5' A-T and 5′ G-T DNAs in the presence of indicated proteins. Recovered products of the MMR reactions were cleaved with AccI, separated in denaturing agarose gels, transferred onto nylon membranes, and hybridized with a ^32^P-labeled probe (5'- ACTCTCAGGCAATGACCTGATAGCC-3′) that is complementary to the discontinuous strand of the 5' A-T and 5′ G-T DNAs. The indirectly labeled products were visualized using a Typhoon phosphorimager. The sketches outline the 5' A-T and 5′ G-T DNAs and indicate relative positions of the ^32^P-labeled probe. The *arrow* marks a location of smallest strand-displacement products that removed the G-T mismatch from the DNA. MMR, DNA mismatch pair; Pol δ, DNA polymerase; Pol ε, DNA polymerase ε.

In addition to 3' heteroduplexes, the MMR system corrects mismatches on 5′ heteroduplexes. We therefore studied if DNA2 and its variants affected mismatch removal on a 5' heteroduplex in the presence of MutSα, MutLα, PCNA, RFC, and RPA. In line with our earlier data obtained using a 3′ heteroduplex DNA substrate (Fig. 3), we found that DNA2 and DNA2-K654R promoted mismatch removal on a 5' heteroduplex in the presence of MutSα, MutLα, PCNA, RFC, and RPA, but DNA2-D277A and DNA2-D277A-K654R did not (Fig. 4*B*, lanes 2–6, and graph). We next asked whether the DNA2-promoted mismatch removal reaction on the 5′ heteroduplex could occur in the absence of the mismatch recognition factor MutSα or MutLα endonuclease. The data showed that the omission of MutSα or MutLα from the reaction mixture did not abolish the DNA2-promoted mismatch removal reaction on a 5' heteroduplex (Fig. 4*B*, lanes 7–8, and graph). This suggested that DNA2 enhanced the MutSα-and MutLα-independent mismatch correction reaction by increasing the strand-displacement activity of Pol δ holoenzyme. Further support for this idea came from an experiment in which we ascertained that DNA2 strongly increased the strand displacement–based mismatch removal on the 5′ heteroduplex by a four-protein system consisting of Pol δ, PCNA, RFC, and RPA (Fig. 5*A*). In addition, we determined that human Pol ε, an enzyme that does not have a significant strand-displacement activity (48, 69), was not able to replace Pol δ in the DNA2-promoted mismatch correction reaction on the 5′ heteroduplex (Fig. 5*B*).

We also utilized a Southern hybridization with a ^32^P-labeled probe to visualize the DNA2-promoted strand-displacement DNA synthesis products that were separated on denaturing agarose gels. The experiments showed that DNA2 enhanced the strand-displacement DNA synthesis activity of Pol δ holoenzyme on the 5' heteroduplex and 5′ homoduplex with a similar efficiency (Fig. 5*C*, lanes 3 and 8), and that MutSα did not affect the DNA2-promoted strand-displacement DNA synthesis on either DNA (Fig. 5*C*, lanes 4 and 9).

We next performed experiments to determine the size of DNA products formed by DNA2 in reconstituted strand-displacement DNA synthesis reactions that occurred in the presence or absence of MutSα. The substrate in these reactions was a 6.4-kb circular ssDNA that was annealed with 13 oligonucleotides, one of which had a base mismatch and was labeled at its 5′ end with ^32^P. The results showed that DNA2 formed 8 to 14 nt products in the strand-displacement DNA synthesis reactions in a MutSα-independent manner (Fig. S3). This finding suggests that when the Pol δ generated an 8 to 14 nt flap during the strand-displacement reaction, it was removed by DNA2.

## Discussion

EXO1 is the only exonuclease that has been shown to excise the mismatch in eukaryotic MMR (45, 50, 52, 53, 70). EXO1 preferentially acts on DNA replication errors formed by Pol α (71), but it is also involved in the correction of mismatches produced by Pol δ and Pol ε (71, 72). Unlike the loss of MutSα or MutLα, the loss of EXO1 does not confer a strong mutator phenotype on yeast and mice (50, 52, 53). This finding indicates that EXO1-independent MMR removes the majority of DNA replication errors when EXO1 is not available. Although significant progress has been made in understanding EXO1-independent MMR (54, 73, 74, 75, 76, 77, 78), this MMR pathway has remained enigmatic.

Prior research revealed that the strand-displacement activity of Pol δ plays a role in EXO1-independent MMR (54, 73, 74). Furthermore, the nucleases FEN1, Rad27, and FAN1 have been implicated in EXO1-independent MMR (75, 76, 77, 78). To address the question of whether there is another player in EXO1-independent MMR, we investigated DNA2 nuclease/helicase, an essential eukaryotic protein that has multiple functions in DNA metabolism (55, 56, 61, 62, 63). We have established that the replacement of *DNA2* with *dna2-P504S* in a WT strain significantly increases the *CAN1* mutation rate (Table 1). Furthermore, we have determined (i) that *dna2-P504S* interacts synergistically with *exo1Δ* for base substitutions, (ii) that *msh2Δ* is epistatic to *dna2-P504S exo1Δ* for base substitutions, and that (iii) *pms1-Q723A* is epistatic to *dna2-P504S* for base substitutions (Table 1). These data represent genetic evidence for DNA2 involvement in EXO1-independent MMR.

Our biochemical experiments have shown that DNA2 promotes a defined EXO1-independent MMR reaction that relies on the strand-displacement activity of Pol δ holoenzyme (Figure 2, Figure 3, Figure 4). Earlier research revealed that DNA2 harbors both nuclease and helicase activities (55, 57) and that the helicase activity of DNA2 remains silent in the presence of the DNA2 nuclease activity (57). Our analysis of the helicase- and nuclease-deficient variants of DNA2 has established that it is the nuclease activity of DNA2 that enhances the EXO1-independent MMR reaction (Fig. 3). Subsequent experiments indicated that the DNA2 nuclease activity increases the efficiency of the EXO1-independent MMR reaction by enhancing strand displacement by the Pol δ holoenzyme (Fig. 5). This finding suggests that removal of ssDNA tails by DNA2 increases the strand-displacement DNA synthesis by Pol δ holoenzyme. Unlike Pol δ, Pol ε does not have a significant strand-displacement activity (48, 69). Our observation that the replacement of Pol δ with Pol ε inactivates the DNA2-promoted MMR reaction (Fig. 5*B*) supports the conclusion that the strand-displacement activity of Pol δ holoenzyme drives mismatch removal in the DNA2-promoted MMR reaction.

MutSα activates mismatch excision by EXO1 (45). The functional MutSα–EXO1 interaction is likely to be driven by the physical contact between the two proteins (45, 52). Although DNA2 and MutSα physically interact with each other (Fig. 2*C*), we have been unable to detect that MutSα increases the DNA2-promoted strand-displacement activity of Pol δ holoenzyme on a mismatch-containing DNA (Fig. 5, *A* and *C*). It might be that our system lacks a factor or a protein modification that enables MutSα to stimulate DNA2 in a mismatch-dependent manner.

A basic feature of the eukaryotic MMR mechanism is that MutLα endonuclease incises the discontinuous daughter strand in a MutSα-, PCNA-, and RFC-dependent manner to afford mismatch correction (38, 39). The endonuclease function of MutLα provides a downstream factor with a window of opportunity to enter the DNA *via* a MutLα-generated strand break to remove the mismatch. Previous research uncovered a role for the strand-displacement activity of the Pol δ holoenzyme in mismatch removal in the absence of EXO1 (48, 54, 73, 74). We have now shown that DNA2 contributes to EXO1-independent mismatch removal by enhancing the strand-displacement activity of Pol δ.

Of importance is an observation that like DNA2, the other two nucleases, Rad27/FEN1 and FAN1, that contribute to EXO1-independent MMR (75, 76, 77, 78) have 5′ flap endonuclease activities (79, 80, 81, 82, 83, 84), and one of them (Rad27) enhances the strand-displacement DNA synthesis by a Pol δ holoenzyme (78). This observation reinforces the view that the strand-displacement activity of the Pol δ holoenzyme plays a key role in EXO1-independent MMR. It will be important to perform quantitative analyses to determine the effects of EXO1, FEN1, Rad27, and FAN1 on the reconstituted DNA2-promoted MMR reaction to better understand the impact of DNA2 on MMR.

## Experimental procedures

### *S. cerevisiae* strains and measurements of the mutation rates

Yeast strains used in this work were the haploid WT strain E134 (*MATa ade5-1 lys2::InsE-A*_*14*_ *trp1-289 his7-2 leu2-3,112 ura3-52*) (53) and its mutant derivatives. The gene knockouts were generated utilizing PCR-amplified disruption cassettes (84) and the lithium/PEG-based transformation method (85). All gene disruptions were confirmed by PCR. The replacement of *DNA2* with *dna2-P504S* allele was performed using the integration-excision method. The presence of the *dna2-P504S* mutation was confirmed by DNA sequencing.

Spontaneous *CAN1* mutation rates were measured using fluctuation tests that were carried out according to a previously described method (86). Briefly, single colonies obtained from 2 to 3 independent isolates of the same yeast genotype were used to start 12 to 24 cultures each in 3-ml YPD medium (1% yeast extract, 2% bactopeptone, 2% dextrose), supplemented with 60 mg/l adenine and 60 mg/l uracil that were grown at 23 °C. Dilutions of the cultures were plated on a synthetic complete medium to score the total number of cells and on a drop-out medium that lacked arginine and contained 60 mg/l L-canavanine to score the total number of *can1* mutants. Colony counts were utilized to calculate the spontaneous *CAN1* mutation rates with the Drake formula (87, 88). Mutation rates are presented as median values with 95% confidence intervals.

### Human proteins

Human MutLα, MutSα, PCNA, Pol δ, Pol ε, RFC, and RPA were isolated in near-homogeneous forms as previously described (48, 54). Human DNA2, DNA2-D277A, DNA2-K654R, and DNA2-D277A-K654R that were flag-tagged at the C termini were each purified from insect Sf9 cells by chromatographies on α-Flag M2 beads (Sigma) and a MonoS column (GE HealthCare). Baculoviruses that carried the codon-optimized *DNA2*, *DNA2-D277A*, *DNA2-K654R*, and *DNA2-D277A-K654R* genes (57) were used for production and purification of DNA2 and its variants.

### MMR and mismatch excision reactions

MMR reactions were carried out at 37 °C in 40-μl mixtures that contained 20 mM Hepes–NaOH (pH 7.4), 5 mM MgCl_2_, 3 mM ATP, 110 mM KCl, 25 μM dGTP, 25 μM dATP, 25 μM dTTP, 25 μM dCTP, 4 mM DTT, 0.2 mg/ml bovine serum albumin, 0.3 μg DNA (a 3′-gapped heteroduplex, a 5′-nicked heteroduplex, or a 5′-nicked homoduplex), and indicated human proteins. When human MutSα, MutLα, PCNA, RFC, RPA, Pol δ, and Pol ε were present in the reaction mixture, their concentrations were 25, 10, 30, 10, 10, 10, and 20 nM, respectively. Human DNA2 and its variants were included in the reaction mixtures at 0 to 30 nM as indicated. The 3′-gapped heteroduplex DNA (7.54 kb) contained a 21-nt gap that was 304 bp 3' to an A-C mispair, the 5′-nicked heteroduplex (6.44 kb) carried a nick that was 128 bp 5′ to a G-T mismatch (70), and the 5' -nicked homoduplex DNA was identical to the 5′-nicked heteroduplex except that it lacked a mispair. Unless noted otherwise, MMR reactions were carried out for 20 min. Mismatch excision reactions were performed exactly as the MMR reactions except that the reaction mixtures lacked the four dNTPs. MMR and mismatch excision reactions were each terminated by the addition of a 30-μl mixture containing 0.31% SDS, 0.36 M NaCl, 12 mM EDTA, 0.3 μg/μl proteinase K, and 1.8 μg/μl glycogen, followed by incubation of the mixtures at 50 °C for 15 min. The mixtures were extracted with phenol/chloroform, and the DNAs from the supernatants were precipitated with isopropanol. MMR on the 3′-gapped heteroduplex was scored by cleavage of the recovered reaction products with BspEI and AlwNI. To score MMR on the 5′-nicked heteroduplex, the recovered reaction products were cleaved with HindIII and ClaI. To determine the level of mismatch excision on the 3′-gapped heteroduplex, the recovered reaction products were digested with HindIII and AlwNI. After cleavage with restriction endonucleases, the recovered reaction products were separated in 1.1% agarose gels in 1x TAE (40 mM Tris-acetate, 1 mM EDTA, pH 8.2) and stained with ethidium bromide. The images were obtained with a cooled charge-coupled device camera (Fotodyne) and the DNA species were quantified using an ImageJ software.

### Coimmunoprecipitation assays

Coimmunoprecipitation assays were performed as previously described (48). Antibodies against human MSH2 (sc-376384, Santa Cruz Biotechnology), DNA2 (sc-393323, Santa Cruz Biotechnology), and MLH1 (sc-271978, Santa Cruz Biotechnology) were used in coimmunoprecipitation assays.

## Data availability

All data are contained within the article.

## Supporting information

This article contains supporting information (67).

## Conflicts of interest

The authors declare that they have no conflicts of interest with the contents of this article.
