# Supplementary material for: The nuclease activity of DNA2 promotes exonuclease 1–independent mismatch repair
Source: J Biol Chem. 2022 Mar 15;298(4):101831. doi: 10.1016/j.jbc.2022.101831 (PMC9036127; doi:10.1016/j.jbc.2022.101831)
Supplement: Supplemental Figures S1–S3 [file mmc1.docx]

**Supporting Information**


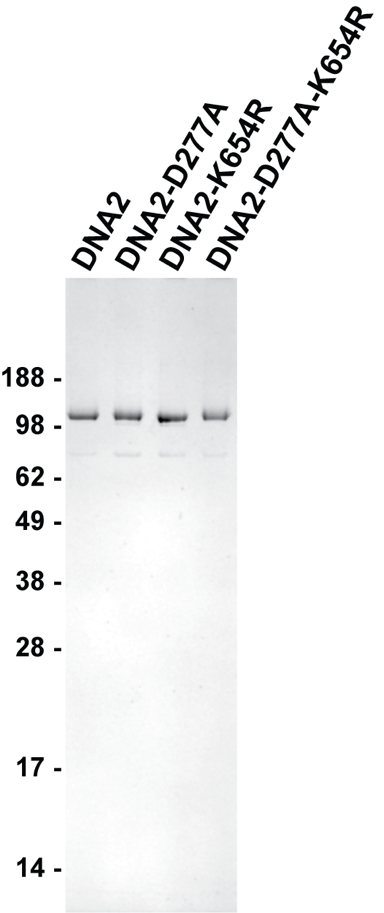


**­**

**Figure S1. Purified DNA2, DNA2-D277A, DNA2-K654R, and DNA2-D277A-K654R that were utilized in this study**. The proteins that were obtained at the final purification step (Experimental Procedures) were separated in an SDS-polyacrylamide gel and visualized with Coomassie blue R-250 staining.

**
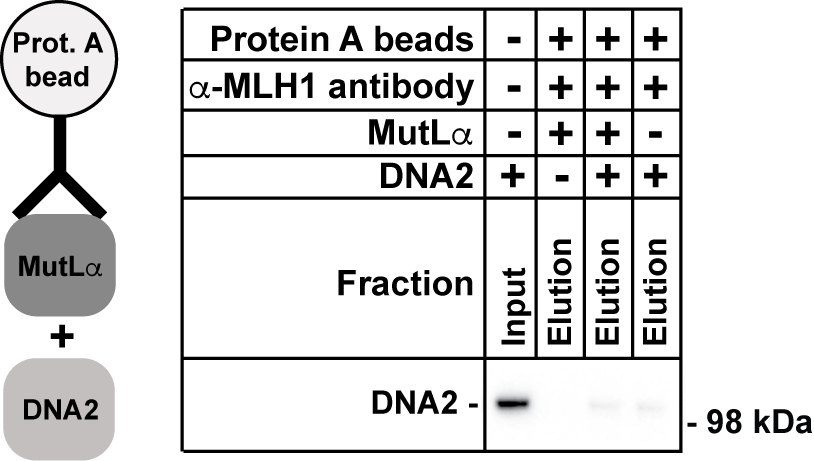
**

**Figure S2**. **DNA2 does not form a stable complex with MutLα in a pull-down assay.** A protein-protein interaction between DNA2 and MutLα was analyzed using a coimmunoprecipitation assay (Experimental Procedures). When indicated, 30-µl reaction mixtures contained MutLα (12 pmol) and DNA2 (3.4 pmol). 0.4% of the input and 25% of the eluted fractions were analyzed by immunoblotting.


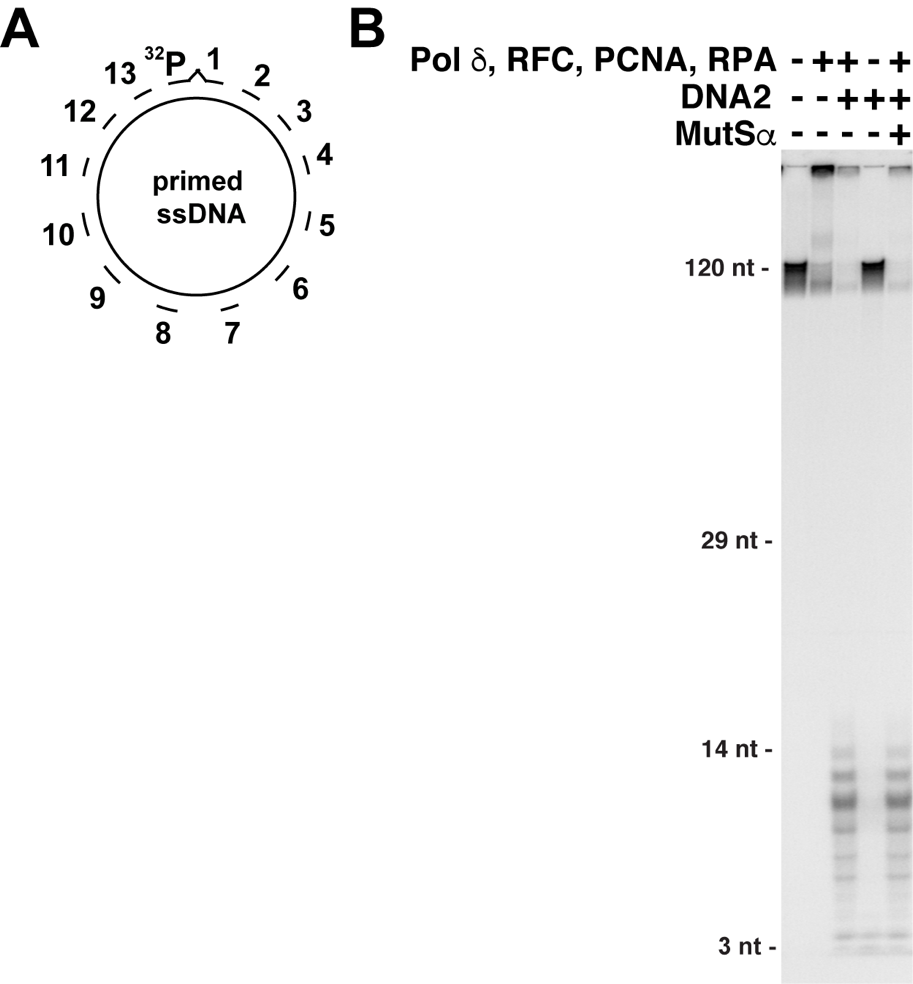


**Figure S3. DNA products formed by DNA2 in reconstituted strand displacement DNA synthesis reactions.** The reconstituted reactions were carried out as described in Fig. 5A except that a ^32^P-labeled primed f1 MR59 ssDNA (0.1 µg) carrying a G-T mismatch was used, instead of 5' G-T DNA, as a substrate. After 10-min incubation at 37°C, the reactions were stopped by the addition of formamide and EDTA to 58% and 13 mM, respectively. DNA products were separated on a 15% polyacrylamide gel containing 6 M urea. After electrophoresis, the gel was dried, and the ^32^P-labeled DNA species were visualized by phosphorimaging. (A) outline of the ^32^P-labeled primed f1 MR59 ssDNA substrate. Oligonucleotide 1 was a gel-purified 120-mer (Sigma) and oligonucleotides 2-13 were 24-mers (IDT). The 120-mer was labeled at its 5' end with ^32^P and carried a base mismatch which formed a G-T mispair when annealed with 6.4-kb f1 MR59 ssDNA (67). The distance between the annealed oligonucleotides 1 and 2, 2 and 3, 3 and 4, 4 and 5, 5 and 6, 6 and 7, 7 and 8, 8 and 9, 9 and 10, 10 and 11, 11 and 12, and 12 and 13 was 500-600 nt, whereas the distance between the annealed oligonucleotides 13 and 1 was 20 nt. The sequences of the DNA oligonucleotides are available upon request. (B) A representative gel image that shows ^32^P-labeled products formed in reactions that were reconstituted in the presence of indicated human proteins.
